# Evaluation of work disability and the international classification of functioning, disability and health: what to expect and what not

**DOI:** 10.1186/1471-2458-12-470

**Published:** 2012-06-21

**Authors:** Jessica Anner, Urban Schwegler, Regina Kunz, Bruno Trezzini, Wout de Boer

**Affiliations:** 1 asim, Academy of Swiss Insurance Medicine, University Hospital Basel, Basel, Switzerland; 2 Swiss Paraplegic Research, Nottwil, Switzerland

**Keywords:** International Classification of Functioning, Disability and Health, Disability evaluation, Handicapped role

## Abstract

**Background:**

Individuals who are sick and unable to work may receive wage replacement benefits from an insurer. For these provisions, a disability evaluation is required. This disability evaluation is criticised for lack of standardisation and transparency. The International Classification of Functioning, Disability and Health (ICF) was developed to express the situation of people with disability. We discuss potential benefits of the ICF to structure and phrase disability evaluation in the field of social insurance. We describe core features of disability evaluation of the ICF across countries. We address how and to what extent the ICF may be applied in disability evaluation.

**Discussion:**

The medical reports in disability evaluation contain the following core features: health condition, functional capacity, socio-medical history, feasibility of interventions and prognosis of work disability. Reports also address consistency, causal relations according to legal requirements, and ability to work. The ICF consists of a conceptual framework of functioning, disability and health, definitions referring to functioning, disability and health, and a hierarchical classification of these definitions. The ICF component ’activities and participation’ is suited to capture functional capacity. Interventions can be described as environmental factors but these would need an additional qualifier to indicate feasibility. The components ‘participation’ and ‘environmental factors’ are suited to capture work requirements. The socio-medical history, the prognosis, and legal requirements are problematic to capture with both the ICF framework and classification.

**Summary:**

The ICF framework reflects modern thinking in disability evaluation. It allows for the medical expert to describe work disability as a bio-psycho-social concept, and what components are of importance in disability evaluation for the medical expert. The ICF definitions for body functions, structures, activity and participation, and environmental factors cover essential parts of disability evaluation. The ICF framework and definitions are however limited with respect to comprehensive descriptions of work disability.

## Background

Individuals who are unable to work because of sickness or injury can receive support for return to work and/or wage replacement benefits if they are unable to return to work. The legal rules require these individuals to file a claim and undergo a medical evaluation of work disability in the field of social insurance (hereafter: disability evaluation). The concept of ‘disability evaluation in social insurance’ itself is equivocal. Literature defines disability evaluation in different ways [1-4]. One way to settle this matter is to analyse the reports of disability evaluation in different countries. Different countries have different ways to organise disability evaluation, but the reports seem to share essential characteristics: Reports describe a claimant’s (in-) capacities and relate these to his health condition (rather than to a non-medical cause) [3,5], and his efforts to recover and return to work [4,6-9].

Critics across Europe have pointed to the lack of quality and transparency of disability evaluation [10-14], and in the last decade, the rehabilitation community has begun to use the International Classification of Functioning, Disability and Health (ICF) to picture the situation of people with disability [15]. The ICF provides “a description of situations with regard to human functioning and its restrictions” and serves as a framework to structure the information in a “meaningful, interrelated and easily accessible way” (ICF p 7) [15]. The ICF concepts and definitions promote standardised reporting of work disability [13,16] which could facilitate comparison of disability evaluation across countries. The authors from one study envision the ICF as an international point of reference for conceptualisation work disability [17]. The question of the application of the ICF to disability evaluation however, remains unanswered, especially since in the frame of social insurance legal equity requires specific reporting [18].

In this article, we will first describe the core features of disability evaluation and the core features of the ICF. Then we address how and to what extent the ICF might be applicable in disability evaluation. We concentrate on the medical reports, as these are better documented than the processes of disability evaluation.

## Discussion

### Comparing the output of disability evaluation across Europe

Despite the wide variation of social insurance systems across Europe and country- specific organization of disability evaluation and differences in structure and size of medical reports, we identified 4 core features of work disability for medical experts [6]: 1) the functional capacity of the claimant; 2) the socio-medical history, including the development and severity of the claimant’s health condition, his/her previous efforts to regain health and return to work, and his/her job and social career; 3) the individual prognosis of work disability; 4) the feasibility of interventions to promote recovery and return to work. These features reflect the “handicapped role“ [19], which refers to the role expectations that exist between a disabled person and those in his social environment when the disabled person is in need of support. In the context of work disability, the „handicapped role“ indicates that the claimant may expect support if a) he/she is long-term unable to do work that society normally expects him to perform, and if his/her b) health condition accounts for this disability, and c) provided he/she makes sufficient effort to undergo treatment and rehabilitation.

Professional guidances on disability evaluation advise the medical expert to draft a holistic picture of the claimant [9,20,21].

The medical report must also follow legal requirements, such as to establish a causal relation between a claimant’s health condition and his/her functional capacity. Lack of motivation or lack of opportunity to find work [18,22] does not suffice as reason for work disability. As a testimony of credibility, a consistent description is required, that incorporates claimant’s impairments, limitations in activity, restrictions in work participation and work disability [7,20,23]. Medical examiners must also provide a general statement about work ability; this can be expressed as a percentage, degree of disability or in working hours. Few countries explicitly require the medical examiners to report separately on the health condition, given that the description of functional capacity covers the health condition implicitly [6,22]. Table 1 summarizes the core features of reports on disability evaluation [6].

**Table 1:** Reporting about work disability in social insurance: a European comparison

**Core features for assessing work (in-)capacity**	**Countries required to report the core features**
1) Functional capacity of the claimant	BE, CH, CZ, DE, FI, FR, GB, IT, NL, NO, SE, SI, SK^1^
2) Health condition (disease, symptoms, complaints)	CH, FI, NL, NO, SE
3) Socio-medical history (claimant's development and severity of ill health condition, his previous efforts to regain health and return to work, job and social career)	BE, CH, CZ, DE, DK, FI, FR, IT, NL, NO, RO, SE, SI, SK
4) Prognosis of work disability (Prognosis of disease and functional capacity)	BE, CH, CZ, DE, FI, FR, GB, IT, NL, NO, RO, SE, SI, SK
5) Feasibility of therapeutic and rehabilitative interventions	BE, CH, DE, FI, FR, GB, IT, NL, NO, RO, SE, SI, SK
6) Causality: functional incapacity exclusively caused by a health condition	CH, DE, FR, NL
7) Consistency between impairments, activity limitations and restrictions in work	CH, DE, NL
8) Ability to work	Expressed as percentage in BE, CH, FR
	Expressed as degree of disability in CZ, NL, SL, SI, RO
	Expressed as hours of work: DE

BE = Belgium, CH = Switzerland, CZ = Czech Republic, DE = Germany, FI = Finland, FR = France, GB = Great Britain, IT = Italy, NL = Netherlands, NO = Norway, RO = Romania, SE = Sweden, SI = Slovenia, SK^1^ = Slovakia. According to international abbreviation: http://www.iana.org/domains/root/db.

### The international classification of functioning, disability and health

In 2001 the World Health Organisation (WHO) adopted the International Classification of Functioning and Disability and Health as a means to describe health, functioning and disability for populations and individuals within health related domains [15], such as rehabilitation [24], statistical analysis [25], education [26], and governance [27]. The ICF is presented as a conceptual framework of disability and health, as well as a hierarchical classification of 1424 coded categories and 1122 definitions. For the purpose of this article, we consider coded categories and definitions separately because coded categories serve for coding and definitions explain the content of the categories.

**Figure 1:**
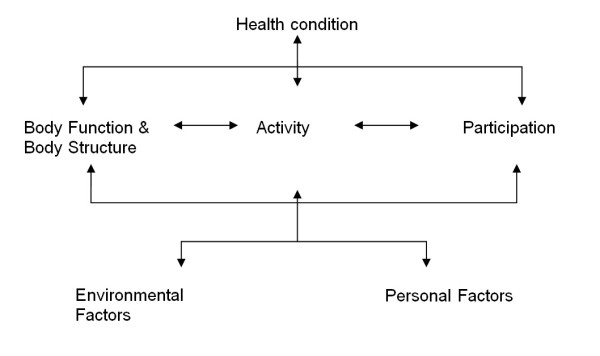
The framework of the ICF.

The ICF framework reflects a bio-psycho-social approach to depict health and disability in different components: health condition, body structure and body function, activity, participation, environmental factors, and personal factors (see Figure 1) [15,28]. Body functions are physiological functions of body systems (including psychological functions). Body structures are anatomical parts of the body such as organs, limbs etc. Activity is the execution of a task or action by an individual and participation is involvement in a life situation. Activity and participation can be described as performance (when considering the real life situation/environment) and capacity (when considering a standardized environment). Environmental factors make up the physical, social and attitudinal environment in which people live and conduct their lives (ICF, p. 10). They can be either a facilitator or a barrier to the individual. Personal factors refer to the particular background of an individual's life and living and comprise features that are not part of a health condition or health states (ICF, p. 17) [15].

**Figure 2:**
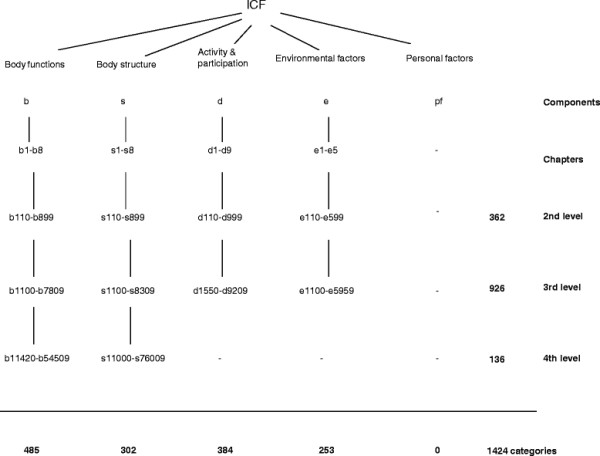
The classification of ICF categories in hierarchical organization of levels.

In the ICF classification, the same components are used (except the health condition) but body structures and body functions are taken apart and activity and participation are taken together. The components, with the exception of personal factors, are subdivided into 1424 categories (Figure 2). Each category is linked to a unique code. 1122 categories (in body functions, activity and participation, and environmental factors) have an explicit definition. Body structures are not defined but mentioned as categories [15]. Qualifiers (no-, mild-, moderate-, severe- and complete problem) can be used to indicate the severity of problems in a category. Table 2 presents an example of an ICF category, its code and definition.

**Table 2:** Example of a code, category and definition

**Code**	**Category**	**Definition**
b280	Sensation of pain	Sensation of unpleasant feeling indicating potential or actual damage to some body structure. Inclusions: sensations of generalized or localized pain in one or more body part, pain in a dermatome, stabbing pain, burning pain, dull pain, aching pain; impairments such as myalgia, analgesia and hyperalgesia

The WHO refrained from classifying personal factors in the ICF classification but researchers have started to propose such definitions to address a perceived gap [29,30]. Figure 2 summarises the alphanumeric structure of the ICF and details of the hierarchical classification.

The ICF framework is widely accepted in rehabilitation, research and policy communities [24]. However, the large number of categories and definitions make it cumbersome to apply the ICF classification in clinical practice and research [31,32]. Disease or setting-specific core sets (e.g. for chronic conditions, acute care, rehabilitation facilities [33,34]) are introduced in order to make using the classification manageable [24].

There is on-going scientific discussion of the precise boundaries and possible shortcomings of the ICF framework or the classification [35-38]. Some of these items of discussion are relevant to our argument:

1. The definitions are connected in a hierarchical fashion that allows specification and aggregation but no other relationships between the definitions, such as causal relationships. This gives the ICF classification the character of a dictionary [39].

2. The dynamic aspect of the development of disability over time is not addressed in the ICF framework or the classification. The descriptions of health or health-related domains represent a given moment while the disability evaluation explores a claimant’s history and prognosis. A line-up of several snap shots of the claimant’s health and health-related domains would be required to indicate the dynamic of the development over time (ICF, p. 220) [15].

### Medical evaluation of work disability in the social insurance and the ICF: bringing the two together

In this section, we discuss as to what degree the current ICF framework, the definitions, and the classification may capture the core features in the reports on work disability (see Table 3).

**Table 3:** Core features in disability evaluation and their coverage in the ICF

**Core features for assessing work (in-)capacity**	**ICF Framework**	**ICF Definitions**	**Remarks**
1) Functional capacity of the claimant	Activity and participation	Activity and participation	
2 Health condition (Disease, symptoms, complaints)	Health condition Body functions/structures	(∅) Body functions/ structure	Disease is a component of the ICF framework but not included in the ICF definitions. It can be coded in the ICD^*^.
3) Socio-medical history (claimant's development and severity of ill health condition, his previous efforts to regain health and return to work, job and social career)	Implicit in the framework but no explicit presentation	∅	The ICF definitions do not cover the development over time.
4) Prognosis of disease and functional capacity	∅	Partly: capacity	The ICF framework and ICF definitions do not cover the time perspective.
5) Feasibility of interventions and rehabilitation	Environmental factors	Environmental factors (facilitators and barriers)	The ICF framework and ICF definitions cover intervention and rehabilitation however; they do not cover dynamic time perspective or the qualification ‘requirement to comply’.
6) Causality: functional incapacity exclusively caused by health condition	∅	∅	The ICF framework displays a person holistically
7) Consistency of the situation of the claimant	Partly: between the impairments, activity limitations and restrictions in work	∅	
8) Ability to work (in general hours and %)	∅	∅	

Legend: ∅ = not part of the ICF framework or the ICF definitions, *ICD: International Classification of Disability.

#### The framework

The ICF framework describes disability as a composite concept that integrates impairments, activity limitations, and participation restrictions with personal and environmental factors. As such, the framework is well suited to present work disability as a particular manifestation of disability. In general, the ICF framework dwells on the interaction of the health condition with the functioning of the individual (rather than on aetiology or disease) [40]. It also visualizes the relevance of environmental and personal factors on all components [23]. Professional guidance to insurance physicians from an increasing number of countries keeps stressing the importance of the benefits of the framework and discourages a traditional biomedical approach that simplifies disability as a specific state of health [20,21,41].

Disability is a process rather than a state. Disability refers to the past, present, and future outcome of a person’s interaction with his/her physical, social, cultural and legislative environment [17]. The ICF framework does not address this process aspect explicitly. The personal factors include aspects of the past (such as education) but in a static way. We are unable to describe the dynamic development of health and health-related domains, nor are there means to express the future events and prognosis of work [38]. With capacity, we can indicate the expected performance in a standardized environment but are still missing the dynamic development. This is a significant limitation of the ICF framework.

In several countries such as such as France [42], Germany [21], the Netherlands [41], and Switzerland [20] restricting the causal relation between the health condition and activities is explicitly requested in order to recognise legal work disability. Limitation of activities resulting from lack of motivation, or lack of participation resulting from unemployment does not count. The ICF framework distinguishes the domains and their interaction but does not foresee a restricted causal relation. The guidance of disability evaluation in these countries encourages the insurance physicians to first draw a holistic picture of the claimant, compatible with the framework and to then discount the non-medical factors from the overall judgement of disability. It is unclear how the ICF framework can capture these aspects of disability evaluation.

#### The definitions

As stated above, the ICF classification contains 1122 explicit definitions (not including body structures or personal factors). The definitions can serve to standardize and harmonise the evaluation reports, and avoid ambiguity and variation in the presentation and interpretation of the findings. Our question is if the ICF definitions capture the core features of disability evaluation.

The core features functional capacity, health state, and the ability to participate in working life can be described with the components ‘body structure/function’ and ‘activity and participation’. As the ICF has not been specifically developed for work disability, it stands to be tested if the present set of definitions is comprehensive in this field.

Aspects of the socio-medical history and prognosis can be depicted with the definitions, but it is not practicable to line up the content in a chronological sequence. Like the framework, the definitions, do not describe the dynamic development of disability. Therefore, socio-medical history, and prognosis are not easily covered in the ICF definitions.

Interventions can be described as facilitating environmental factors. In disability evaluation, we need to qualify some interventions as feasible. Such qualifiers do not exist currently, which stresses the need to develop them within the ICF concept of environmental factors.

Further, disability evaluation gives a judgment on the claimant's situation. This can be given from two different viewpoints: the (self-) perception of the claimant and the perception of the medical expert. Medical experts usually integrate both perceptions in their reports. Applying the ICF would make it necessary to keep the two systematically apart. Although it is no difficult to separate the two and it can be considered beneficial to do so, it is not a common practice.

Restricting the cause why a person is not able to work is an important statement in disability evaluation. The ICF definitions cannot describe causal relation because the current ICF definitions cannot be put together.

Finally, medical examiners must also provide a general statement about work ability. Percentage, degree of disability or in working hours cannot be described with ICF definitions

#### The classification

The classification organises categories and definitions in a hierarchical system. The applicability of the classification goes as far as the application of the definitions goes. The refined coding system of the ICF classification can be useful in research, or for documentation, or in the statistics of a social insurance administration. For these purposes core sets have been published in the field of disability evaluation as well. These core sets facilitate the description of functional capacity [16,43]. For the other core features different core sets could be developed.

Overall, we feel that using the ICF for development of disability evaluation does hold promises but it also has its limitations. The ICF framework fits modern thinking about disability evaluation. It helps medical experts to describe work disability as a bio-psycho-social phenomenon rather than as biomedical phenomenon only. The framework illustrates the connections between the different components in the disability evaluation that the medical expert has to address. The ICF definitions for body functions, structures, activity and participation, and environmental factors cover essential parts of the disability evaluation. Empirical testing is needed to establish if the definitions are useful and sufficiently detailed. Clear and broadly accepted definitions will support the understanding of the medical reports for professionals and administration and allow the development of instruments.

The ICF framework and definitions are limited in the following aspects: the dynamism of development of disability, definitions for personal factors and, causality and consistency. An explicit time dimension could supplement the present ICF framework. Describing “history and prognosis” in words may overcome the lack of dynamic time perspective. For feasibility of interventions qualifiers could be developed.

Empirical research would be needed to test our considerations in practice. Several studies are underway. In one study, we are testing the consensus-based 20-item core set for functional capacity suggested by the European Union of Medicine in Assurance and Social Security (EUMASS) [16] for applicability and usefulness across several European social insurance systems.

In another study, Kirschneck et al. translated concepts of disability evaluation to ICF categories by linking medical reports from claimants with low back pain and chronic widespread pain and compared them with the existing core set of these conditions [13]. The preliminary results of the study show consistency between the pre-existing core sets and the medical reports in Germany [44].

In a third study, we tested the potential of applicability the ICF core sets of low back pain and chronic pain in disability evaluation in Switzerland [45]. We studied 72 medical reports from claimants with low back pain/chronic widespread pain and linked those to the ICF categories.

In a fourth study, Linden et al. have tested an ICF-based instrument to assess functional incapacity in patients with mental health problems [46]. The instrument probes on 13 items of the ICF-component ‘activity and participation’ that are commonly affected in patients with mental disease (e.g. endurance or self-assertiveness).

## Summary

We determined how and to what extent the ICF could capture the medical reports of disability evaluation by defining the key aspects of the disability evaluation and relating them to the framework and the definitions of the ICF.

When evaluating work disability, the medical expert describes the claimant‘s health condition and functional limitations, socio-medical history, feasible interventions and prognosis and relates his/her findings to the requirements of the social insurance scheme. The ICF framework seems to reflect the view of the modern medical expert, especially with regard to functional capacity. However, the framework does not incorporate certain critical elements of a disability evaluation such as the dynamic time perspective or the restricted causal connection between functional capacity and the health condition. The ICF definitions enable the medical expert to report systematically about health aspects and actual functional capacity, and to a lesser extent, work characteristics. The ICF might provide useful concepts and definitions, especially in countries where medical examiners do not describe functional capacity in a structured manner [6].

Before advancing with applied research around the optimal use of the ICF in disability evaluation, the professional community needs to specify its expectations: in what way should the ICF framework and the classification be used to express a claimant’s functional capacity? How could the application of the ICF improve the medical report? What additional benefit would an ICF-based functional capacity assessment provide to the professionals who perform the disability evaluation, to the administrators in the social insurances who use the results, and to researchers who want to support disability evaluation with evidence? On-going research indicates the potential of the ICF to express functional capacity in disability evaluation.

## Competing interests

The authors declare that they have no competing interests.

## Authors' contributions

Jessica Anner and Wout de Boer prepared the first draft of this paper. The other authors have made substantial comments on the content of this manuscript. All authors read and approved the final manuscript.

## Pre-publication history

The pre-publication history for this paper can be accessed here:

http://www.biomedcentral.com/1471-2458/12/470/prepub
